# Supplementary material for: Subcellular protein localisation of Trypanosoma brucei bloodstream form-upregulated proteins maps stage-specific adaptations
Source: Wellcome Open Res. 2023 May 23;8:46. Originally published 2023 Jan 31. [Version 2] doi: 10.12688/wellcomeopenres.18586.2 (PMC10209625; doi:10.12688/wellcomeopenres.18586.2)
Supplement: Peer review history

# Peer review: Reviewer response for version 2

**DOI:** 10.21956/wellcomeopenres.21501.r58162

**Published:** 2023-06-05

**Authors:** Alena Zikova

**Affiliations:** 1 Biology Centre, Institute of Parasitology, Czech Academy of Sciences, České Budějovice, Czech Republic

I would like to thank the authors for considering the reviewers' suggestions. They have done their best, and I have no further reservations or requests.

Is the work clearly and accurately presented and does it cite the current literature?

Partly

If applicable, is the statistical analysis and its interpretation appropriate?

I cannot comment. A qualified statistician is required.

Are all the source data underlying the results available to ensure full reproducibility?

No

Is the study design appropriate and is the work technically sound?

Yes

Are the conclusions drawn adequately supported by the results?

Yes

Are sufficient details of methods and analysis provided to allow replication by others?

Yes

Reviewer Expertise:

Trypanosoma biology

I confirm that I have read this submission and believe that I have an appropriate level of expertise to confirm that it is of an acceptable scientific standard.

# Peer review: Reviewer response for version 2

**DOI:** 10.21956/wellcomeopenres.21501.r58161

**Published:** 2023-05-24

**Authors:** Christine Clayton

**Affiliations:** 1 Centre for Molecular Biology (ZMBH), Heidelberg University, Heidelberg, Germany

This is now considerably improved, though I still think that the data aren't as accessible as they could be. The "how-to" giude in Zenodo is more useful though it still requires some computing expertise (ability to use line command) to do all but download tables. I now see where the localizations are on the Table (which was requested by all reviewers) but getting the microscopy would be pretty complicated. I'm afraid many people won't bother trying, which is a pity.

Is the work clearly and accurately presented and does it cite the current literature?

Yes

If applicable, is the statistical analysis and its interpretation appropriate?

I cannot comment. A qualified statistician is required.

Are all the source data underlying the results available to ensure full reproducibility?

No

Is the study design appropriate and is the work technically sound?

Yes

Are the conclusions drawn adequately supported by the results?

Yes

Are sufficient details of methods and analysis provided to allow replication by others?

Partly

Reviewer Expertise:

Trypanosome gene expression

I confirm that I have read this submission and believe that I have an appropriate level of expertise to confirm that it is of an acceptable scientific standard.

# Peer review: Reviewer response for version 1

**DOI:** 10.21956/wellcomeopenres.20611.r55329

**Published:** 2023-03-20

**Authors:** Michael D. Urbaniak

**Affiliations:** 1 Biomedical and Life Sciences, Faculty of Health and Medicine, Lancaster University, Lancaster, England, UK

The Tryptag project that has generated images of mNeon-green tagged protein in procyclic form (PCF) *Trypanosoma brucei* has been immensely useful in determining protein sub-cellular localisation and by inference function. In this work the authors have extended the project to include the clinically relevant bloodstream form (BSF) for selected protein sets, with a focus on those that are BSF enriched at transcript level or did not generate distinct signal in the PCF, in addition to certain nuclear proteins. This is a laudable effort and I have no doubt that it has been performed to the highest technical standards and will be of great utility to the community. However, the overall presentation of the data is disappointing and format of the available underlying data is unsuitable.

 The manuscript is rather short, which as a data-set paper would be fine, but the brevity extends to a lack of specific details in the results and discussion. There are several times that we are told the rather vague ”many” without the precision of hard numbers, percentages or readily available tabulated data to back up these statements. Identifying the results that support the assertions made should be made clearer and not requires a deep dive into the underlying data.

 I find the format of the data presented in Figure 2 & 3 hard to interpret visually and would rather the data were tabulated for clarity, with additional supplementary table identifying which gene have these characteristics i.e. Set 1 “BSF upregulated” of 289 gene ~75% appear to match the threshold (>2.5-fold, P > 0.05) but their identity is unclear.

 In the discussion regarding the differences in localisation of ~15% of proteins between PCF and BSF there is a statement that a “weak cytoplasmic signal” is simply background and “cytoplasm, nuclear lumen and flagellar cytoplasm localisation” is observed in Pcf for unfused mNG. It would be useful to see examples images of this type (in comparison to “genuine” cytoplasmic signal) and to have greater clarity as to which proteins these caveats relate to. The localisation of unfused mNG in BSF should also be presented and discussed as this is a critical control.

 The Bsf upregulated proteins with higher fluorescent signal in BSF than PCF “includes many novel or uncharacterised proteins localising to many different organelles”. The identity and localisation of these proteins should be clearly given in a supplementary table.

Underlying data is opaque

 Whilst all the underlying data is technically available the data provided in the Zendo link is difficult to navigate or gain any sort of high-level view of the data without investing time and effort. Combined with the lack of detail in the manuscript itself, it is very difficult to see summary data (i.e. a list of proteins tagged and whether the localisation could be determined), let alone compare with previous PCF tagging. Simply browsing images is difficult to the point of being technically challenging and most likely beyond everyone except the most dedicated and data-savvy.

 It is essential that the authors provide summary data is a user-friendly format that can be easily viewed and interpreted by the casual reader to ensure maximum dissemination and impact of the work. I would also urge the authors to integrate these results into the existing Tryptag resource so that they are searchable and can be readily viewed, and to work to integrate them into the TritypDB functional genomic database.

**Minor points:**

1. The introduction states “However, genome-wide mapping of the global changes are broadly limited to gene expression level, most extensively determined at the mRNA level ^7– 11^ which does not correlate fully with protein abundance ^12^. Few studies consider later steps in protein production: translation (mRNA ribosome footprinting) ^13^ and protein abundance (quantitative proteomics) ^14^. “
  This is not an accurate reflection of the literature, as there are three SILAC quantitative proteomic studies alone that compare Bsf and Pcf protein abundance (PMID: 23090971 ^1^ and references 14 & 26).
2. Reference 13 appear to be erroneous – it is certainly not an mRNA ribosome footprinting study.
3. The language in the manuscript would benefit from minor changes to grammar to ensure clarity, particularly in the abstract and introduction, i.e:
  “Results: We have confirmed the localisation of known [stage-specific proteins ] and identified the localisation of novel stage-specific proteins.”
  “This showed how informative localisation can be for holistic mapping of potential protein function, although naturally [localisation] does not determine specific molecular function.”
  “We also previously used high throughput tagging [of ] BSF-upregulated genes to identify ESB1”

Is the work clearly and accurately presented and does it cite the current literature?

Yes

If applicable, is the statistical analysis and its interpretation appropriate?

Yes

Are all the source data underlying the results available to ensure full reproducibility?

No

Is the study design appropriate and is the work technically sound?

Yes

Are the conclusions drawn adequately supported by the results?

Partly

Are sufficient details of methods and analysis provided to allow replication by others?

Yes

Reviewer Expertise:

Trypanosome cell signalling and post-transcriptional regulation

I confirm that I have read this submission and believe that I have an appropriate level of expertise to confirm that it is of an acceptable scientific standard, however I have significant reservations, as outlined above.

## References

[1] : Comparative proteomics of two life cycle stages of stable isotope-labeled Trypanosoma brucei reveals novel components of the parasite's host adaptation machinery. *Mol Cell Proteomics* .2013;12(1) : 10.1074/mcp.M112.019224 172-9 10.1074/mcp.M112.019224 23090971PMC3536898

# Peer review: Reviewer response for version 1

**DOI:** 10.21956/wellcomeopenres.20611.r54787

**Published:** 2023-03-01

**Authors:** Alena Zikova

**Affiliations:** 1 Biology Centre, Institute of Parasitology, Czech Academy of Sciences, České Budějovice, Czech Republic

No one doubts the importance of genome-wide localization studies. We can clearly see their power in the impact of the TrypTag project ^1^, which was finally published in Nature Microbiology, but whose resources we have long been able to use via the TrypTag or Tritrypdb platforms. Since the whole genome-scale analysis of subcellular localization was performed on the procyclic forms (PCF), one of the two easily cultured forms of *T. brucei*, it is obvious that future studies will also focus on the second form, the bloodstream stage (BSF).

 The authors made a good selection of the initial sets of proteins of interest, namely i) genes that are upregulated (2.5 times, p< 0.05 according to the study by Jensen *et al*, & some others), ii) genes for which no localization in PCF was shown, and iii) genes for which nucleoplasmic or nucleolar localization was shown (actually, a rationale for this third group should be included, as it must not be obvious to many, including me, I just conclude that it is related to VSG).

 Based on the localization data obtained, the authors identify organelles that are subject to stage-specific regulation. They also conclude that in most cases localization does not change when known for both life cycle stages, and that if it was not possible to localize the protein to PCF, it is most likely a BSF protein whose localization can be detected in these forms.

 Overall, the manuscript is quite short, but one was more looking forward to the supplement to evaluate the study in general and for specific IDs. However, I cannot evaluate the supplemental data available from Zenodo. I downloaded everything, clicked on everything, but I did not see any complete tables or images. After opening the TrypTag website, I was sad to see that the data from this study is not yet available on this website.

 I have two main criticisms, which I will address below: i) some vagueness of the text, 2) the lack of supplements and tables to support the figures shown.

 Ad1) - the whole text is interspersed with statements like:

 'we observed convincing fluorescent signal in BSF for many tagged proteins [link to Fig 3B, which is firstly in percentages, so you have to do the math, and secondly it is not even clear how many genes are 100%]';

 '…for many in Set 2...',

 '…many of these genes encode...'

 '...it includes many novel or uncharacterized proteins...'

 One wonders how many is "many." Since the data sets were very discrete, I do not see why the authors cannot be more specific. The same is true for the cut-off for genes up-regulated in BSF, it is only mentioned once that the cut-off is 2.5 with a p-value < 0.05.

 This certain ambiguity relates to Figure 2 and 3, which are a mystery to me. Example - Figure 2A, where the y-axis is in percent and labeled as BSF upregulated proteins - the first column - 8171 is 100% (I guess), so I conclude that 10% are upregulated BSF - that is about 800 proteins, but the authors selected 289 genes..., the second column - 289 is 100%, if so I do not know what this column is supposed to show, and the same is true for column 3 and 4.

 It would be nice if the authors could redesign the diagrams to make them easier to understand (but it is possible that only I have this problem). Nonetheless, I would definitely recommend accompanying these graphs with supplemental Excel spreadsheets highlighting the gene IDs, including the gene description, the determined localization in BSF and PCF.

 This will also help in determining for how many hypothetical proteins subcellular localizations were found. The authors mention this in the abstract, "we have confirmed the localization of known and identified the localization of novel stage-specific proteins" but practically, there is no table or graph (how many and where they are) for this result.

 Figure 1 - I would recommend putting the number of genes in each group in parentheses.

 Ad2) Lack of supplements and tables that would support the figures shown - this was mentioned above, but I would add that a table would also be useful for Figure 5. While it is nice to see the specific organelle adaptation, scientists will always want to look for their favorite gene IDs.

 This is also related to the question of whether it is possible to make the data available on TrypTag; I understand that linking to Tritrypdb will take some time.

 Last but not least, the phenomenon of dissimilar localization between PCF and BSF is very interesting. The authors claim that this is the case in 15% of the genes studied, which is not low. A list of their IDs and a comparison of localization would be very interesting. It is a pity that the authors did not take the published examples as proof of concept, I know of at least two examples - MCP 6 (PCF mito, BSF glycosomes, Colasante *et al.*, 2006 ^2^) and alpha-ketoglutarate decarboxylase (PCF mito, BSF glycosome, Sykes et al., 2015 ^3^).

 Otherwise, I believe that all the supplementary information (list of genes, their descriptions, localizations, and IFA images) is probably available via the files uploaded to Zenodo, but for students/researchers who do not know how to use a programming language, this information is actually inaccessible and reduces the impact of the study on the scientific community. An addition of a detailed description on how to dissect these data would help.

Is the work clearly and accurately presented and does it cite the current literature?

Partly

If applicable, is the statistical analysis and its interpretation appropriate?

I cannot comment. A qualified statistician is required.

Are all the source data underlying the results available to ensure full reproducibility?

No

Is the study design appropriate and is the work technically sound?

Yes

Are the conclusions drawn adequately supported by the results?

Yes

Are sufficient details of methods and analysis provided to allow replication by others?

Yes

Reviewer Expertise:

Trypanosoma biology

I confirm that I have read this submission and believe that I have an appropriate level of expertise to confirm that it is of an acceptable scientific standard, however I have significant reservations, as outlined above.

## References

[1] : Genome-wide subcellular protein map for the flagellate parasite Trypanosoma brucei. *Nat Microbiol* .2023; 10.1038/s41564-022-01295-6 10.1038/s41564-022-01295-6 36804636PMC9981465

[2] : Characterization and developmentally regulated localization of the mitochondrial carrier protein homologue MCP6 from Trypanosoma brucei. *Eukaryot Cell* .2006;5(8) : 10.1128/EC.00096-06 1194-205 10.1128/EC.00096-06 16896205PMC1539146

[3] : The krebs cycle enzyme α-ketoglutarate decarboxylase is an essential glycosomal protein in bloodstream African trypanosomes. *Eukaryot Cell* .2015;14(3) : 10.1128/EC.00214-14 206-15 10.1128/EC.00214-14 25416237PMC4346564

# Peer review: Reviewer response for version 1

**DOI:** 10.21956/wellcomeopenres.20611.r54582

**Published:** 2023-02-03

**Authors:** Christine Clayton

**Affiliations:** 1 Centre for Molecular Biology (ZMBH), Heidelberg University, Heidelberg, Germany

Results from the Tryptag project constitute an immensely valuable resource. The ability to select proteins localized to particular structures or sub-structures within the trypanosome has already resulted in several publications and greatly facilitates interpretation of various types of data - such as distinguishing interaction partners identified by mass spectrometry from likely contaminants. A gap, however, was that the project was done using the procyclic form. This meant that N-terminally tagged proteins that are expressed exclusively in the bloodstream form could not be detected. Since mRNA regulation usually relies on 3'-untranslated regions, tagging at the C-terminus (which also replaces the 3'-UTR) might solve that issue but could also result in aberrant expression. In this manuscript the authors now describe localizations for bloodstream-form-specific proteins, with an emphasis on N-terminal tagging, except where N-terminal signal sequences were expected.

 The paper is fine as far as it goes but currently of limited use because I found it impossible to access any of the underlying data, which are currently available only via a (to me, impenetrable) Zenodo folder. This was incredibly frustrating, I couldn't even see which genes had been analyzed.

 There are two important gaps that must be filled:

1. The new localizations do not appear to be available via the Tryptag web site. I found only one image set for Tb927.11.1230, for example. The data must be uploaded onto Tryptag, unless there is a compelling reason why this is not possible. There should also be links to TritrypDB.
  I am not a total idiot with regard to code but after following the Zenodo link (and downloading the zipped folder) I hadn't a clue what to do next. If it really is possible to get the data this way, it is essential to provide a really clear guide suitable for people who are not able to deal with code at all. But uploading onto Tryptag would be way preferable.
2. The paper must include a supplement which is simple spreadsheet listing the genes analysed, success of tagging, new results and previous PCF results. The authors presumably have this list since it would be needed to generate their figures. At the same time perhaps the authors could provide such a spreadsheet for the entire procyclic form dataset, since at present it is possible to interrogate (and download from) the Tryptag database only with individual genes or localizations. While I appreciate that no-one should undertake further experiments on a protein without consulting the original images, broader analyses absolutely require these spreadsheets.

Is the work clearly and accurately presented and does it cite the current literature?

Yes

If applicable, is the statistical analysis and its interpretation appropriate?

I cannot comment. A qualified statistician is required.

Are all the source data underlying the results available to ensure full reproducibility?

No

Is the study design appropriate and is the work technically sound?

Yes

Are the conclusions drawn adequately supported by the results?

Yes

Are sufficient details of methods and analysis provided to allow replication by others?

Partly

Reviewer Expertise:

Trypanosome gene expression

I confirm that I have read this submission and believe that I have an appropriate level of expertise to state that I do not consider it to be of an acceptable scientific standard, for reasons outlined above.
